# Different effects of abnormal mechanical stress on temporomandibular joint cartilage, subchondral bone, and discs

**DOI:** 10.3389/fphys.2025.1539342

**Published:** 2025-04-03

**Authors:** Miao Xiao, Shilei Ni

**Affiliations:** The First Department of Oral and Maxillofacial Surgery & Oral Plastic and Aesthetic Surgery, Hospital of Stomatology, Jilin University, Changchun, China

**Keywords:** temporomandibular joint, cartilage, subchondral bone, disc, mechanical stress

## Abstract

**Background:**

Temporomandibular joint disorder (TMJD) is a group of diseases occurring in the temporomandibular joint (TMJ) with clinical manifestations of pain in the joint area, mastication disorders, and restriction of mouth opening, which is one of the most common diseases of the oral and maxillofacial region, and its specific etiology has not yet been fully elucidated. As a biomechanical orchestrator, the TMJ mediates dynamic transduction of masticatory forces during the functional loading cycle. Notably, as a secondary cartilage type, the condylar cartilage exhibits postnatal remodeling that is critically dependent on functional mechanical stimulation. Abnormal mechanical stimulation can result in structural dysfunction of the TMJ.

**Objective:**

The purpose of this review was to outline the remodeling responses of TMJ cartilage, subchondral bone, and disc to abnormal mechanical stimulation of different types and intensities, especially the subchondral bone and articular disc.

**Conclusion:**

Abnormal mechanical stress induces degeneration of the condylar cartilage, characterized by dysregulated chondrocyte proliferation and differentiation, elevated cell apoptosis, and ECM injury. The ability of TMJ condylar cartilage to adapt to changes in the mechanical load environment for remodeling is influenced by age, as well as the type, intensity, and duration of the applied mechanical load. Bone loss is often the first response of subchondral bone to abnormal mechanical forces. Abnormal mechanical stimuli affect nutrient supply and matrix metabolism of TMJ discs.

## 1 Introduction

Temporomandibular joint disorder (TMJD) encompasses a spectrum of musculoskeletal and neuromuscular conditions affecting the masticatory muscles, temporomandibular joint (TMJ), and associated structures. Characterized by cardinal clinical manifestations including joint sounds, localized pain, and restricted mandibular mobility, these disorders significantly impair masticatory function and diminish quality of life (Valesan et al., 2021). The pathogenesis of TMJD is very complex and has yet to be fully clarified. The TMJ mediates dynamic transduction of masticatory force, whose growth, development, and maintenance of tissue function depend on appropriate mechanical force stimulation, and it can be restructured in response to mechanical force stimulation such as tensile, shear, and compressive stress (Liu et al., 2024). Based on the biomechanical characteristics of the TMJ, some scholars have proposed that changes in mechanical stress are closely related to the structural and functional disorders of the TMJ, and studying the biomechanical behavior of the TMJ is of great significance for understanding the pathogenesis of TMJD (Willems et al., 2014; Sobue et al., 2011).

Unlike most joints, the cartilage covering the surface of the TMJ is fibrocartilage, and the extracellular matrix of chondrocytes is mainly composed of proteoglycans and collagen fibers. Collagen fibers are distributed in different areas of fibrocartilage, with type I and III collagen in the surface layer and type II and X collagen in the hypertrophic layer, which are interwoven and arranged into three-dimensional structures to provide tensile, shear, and compressive strength for TMJ (Kuroda et al., 2009). Many scholars have used animal models to study the pathological changes in the structure of the TMJ under different types of abnormal mechanical force stimulation (Ramirez-Yañez et al., 2004; Kaul et al., 2016; etti et al., 2018). In pathological conditions, such as TMJ osteoarthritis, it is characterized by progressive destruction of articular cartilage and subchondral bone resorption (Zhu et al., 2024). Condylar resorption is pathologically characterized by progressive flattening of the articular surface secondary to superficial bone resorption, accompanied by trabecular thinning, decreased bone volume fraction, and a marked reduction in bone mineral density (Zarour et al., 2020; Xiao et al., 2024). Cartilage, subchondral bone, and joint disc can all transmit mechanical stress distributed in TMJ, and the change of mechanical loading mode can cause cartilage degeneration, reduction or increase in subchondral bone trabeculae, and variations in the disc thickness (Pan et al., 2009; Zhang et al., 2018; Yasuno et al., 2024).

At present, there are reviews summarizing the animal models of abnormal mechanical stimulation and the functions of key signaling molecules under different magnitudes of mechanical stimulation. However, there is no review outlining the remodeling responses of TMJ cartilage, subchondral bone, and disc to abnormal mechanical stimulation of different types and intensities, especially the subchondral bone and articular disc. In this review, we sum up the different effects of abnormal mechanical stress on TMJ cartilage and subchondral bone as well as the adaptive response of the disc, to better understand the pathological changes and mechanisms of TMJ dysfunction under mechanical stimulation**.**

## 2 Abnormal mechanical stress induces cartilage degeneration

The chondrocytes in mandibular condylar cartilage are at different stages of maturation, and the cells in the shallow layer have low differentiation and active proliferation, providing cell reserves for the hypertrophic layer below to supplement the reduction of mature cells caused by endochondral osteogenesis (Hunziker et al., 2002). The condylar cartilage may undergo pathological remodeling as a result of a change in mechanical stress, causing abnormal cell proliferation, excessive apoptosis, imbalance in extracellular matrix synthesis and decomposition, and ultimately leading to joint degeneration (Ramirez-Yañez et al., 2004; Zhao et al., 2022).

### 2.1 Enhanced differentiation of chondrocytes and suppressed proliferation

After mice were forced to open their mouths passively using a homemade spring, Raman et al. noticed a significant increase in the chondrocyte differentiation markers BMP2, ColX, and Sox9 (Kaul et al., 2016). Utreja et al. also observed an increase in the expression of ColX by establishing a forced-open model (Utreja et al., 2016). Excessive mechanical compression led to accelerated chondrocyte differentiation. Studies have found that reducing chewing loading by feeding powdered or liquid diets to mice and rats can weaken chondrocyte proliferation (Chen et al., 2009; Pirttiniemi et al., 2004; Kato et al., 2015). The lack of appropriate occlusal force stimulation during growth hinders the normal development of the TMJ condyle. Furthermore, studies have demonstrated that a soft diet suppresses chondrocyte proliferation in newly weaned rabbits, while reduced masticatory function in the soft-diet group not only decreased cartilage thickness but also modulated Notch1 expression (Yan et al., 2021). It has been reported that Notch1 signal transduction is connected to variations in mechanical stress and can serve as a mechanical force sensor in endothelial cells (Mack et al., 2017). The notch signaling pathway is a highly conserved pathway in multicellular organisms that determines cell fate and is closely related to the proliferation and differentiation of chondrocytes and the metabolic balance of the cartilage matrix (Pakvasa et al., 2020; Brou et al., 2000; Mumm et al., 2000). Some studies have suggested that Notch1 signaling may be activated in TMJ osteoarthritis, and activated Notch1 directly regulates the upregulation of ADAM8 expression through Hes1 to promote the degradation of the extracellular matrix of chondrocytes (Duan et al., 2019). In human chondrocytes, shear force can activate Notch1 and immediately induce upregulation of inflammatory cytokines IL-8 and TNF-α (Cheng et al., 2020). These findings suggest that appropriate mechanical loading is essential for activation of Notch1 signaling. The pathophysiological mechanism of TMJ remodeling triggered by mechanical stress is still unclear, and Notch1 is anticipated to emerge as a novel molecular target for investigation.

Cartilage homeostasis is regulated by a complex metabolic pathway network. Wnt/β-catenin is a crucial signaling pathway regulating chondrocyte proliferation and hypertrophy, and its signal dysfunction is also connected to the pathological process of TMJ cartilage injury caused by abnormal mechanical forces (Lu et al., 2022). Cai et al. found osteoarthritis-like changes in TMJ cartilage and activated the Wnt/β-catenin signaling pathway while downregulating the expression of pathway inhibitors SFRP3 and SFRP4 in both the established *in vivo* model of increased vertical distance of modified occlusion and *in vitro* model of chondrocytes subjected to mechanical stress (Cai et al., 2022). Studies have shown that SFRPs are not only an important regulator of the Wnt/β-catenin signaling pathway but also may reduce bone loss and inhibit inflammation to alleviate cartilage degradation (Maekawa et al., 2017). Furthermore, severe compression can contribute to the TMJ’s inflammatory response, which lowers chewing efficiency and can also cause osteoarthritis-like changes in the dysfunctional TMJ (Ikeda et al., 2014; Phero et al., 2021). Therefore, the loading on the TMJ should be considered in the selection of treatment options, such as orthodontics and orthognathic surgery, to achieve good treatment effects while minimizing damage to the TMJ.

### 2.2 Chondrocyte death in cartilage

According to research, reduced occlusal loading by feeding a soft diet resulted in a significant increase in chondrocyte death (Robinson et al., 2019). Zhu et al. made a hooked resin ball on the mandibular incisor of rats and used rubber bands to apply quantifiable compressive mechanical force with the help of the neck and arms, with results showing an increase in chondrocyte apoptosis and a decrease in cartilage thickness (Zhu et al., 2016). The endoplasmic reticulum, as the main storage site for Ca^2+^, can induce endoplasmic reticulum stress (ERS) in cartilage under mechanical pressure. Endoplasmic reticulum Ca^2+^ release mediated by ryanodine receptors RyRs and inositol trisphosphate receptors InsP_3_Rs is involved in mechanical stress-regulated ERS and subsequent chondrocyte apoptosis (Zhu et al., 2016; Li et al., 2013). Lv et al. developed a novel bidirectional microscope loading device to record the transient Ca^2+^ response in chondrocytes under compressive mechanical load, and found that pressure loading significantly promoted Ca^2+^ signal transduction in chondrocytes and increased extracellular Ca^2+^ influx (Lv et al., 2018). Ca^2+^ plays an important role in the mechanical force signal transduction in chondrocytes.

### 2.3 Matrix degradation in cartilage

Aberrant mechanical load induced extracellular matrix (ECM) degradation by upregulating catabolic enzymes (e.g., matrix metalloproteinases (MMPs) and aggrecanases), leading to the disruption of proteoglycan and collagen networks (Huang and Wu, 2010). Chondrocytes may respond to liquid diets by rapidly reducing the secretion of extracellular matrix (Uekita et al., 2015). Yang et al. found that overloading resulted in upregulation of MMP13 and downregulation of collagen type II in cartilage (Yang J. et al., 2020). Zhu et al. also found that excessive compressive force caused high expression of IL-1β and MMP3 (Du et al., 2020). When under abnormal shear stress, the TMJ cartilage underwent damage and irreversible deformation (Kuroda et al., 2009). Some scholars have found that fluid shear stress (SS) greater than 10 dyne/cm2 can cause TMJ chondrocytes to highly express cartilage degeneration markers such as MMP3, MMP13, and IL1-β (Xu et al., 2024; Zhang M. et al., 2019). Wilson et al. found that excessive shear strain along the alignment of collagen fibers can lead to cartilage damage (Wilson et al., 2006). The shear and friction generated during mandibular movement put the condylar cartilage in a tensile state, in which the viscoelasticity of the condylar cartilage is mainly controlled by the solid component (collagen), providing tensile strength for the cartilage (Singh and Detamore, 2009). Therefore, the mandibular condylar cartilage has sufficient stiffness to withstand shear loads, while the destruction of collagen fibers caused by abnormal shear force can reduce the ability of the cartilage to resist abnormal mechanical loads. High fluid shear stress (10–20 dyne/cm^2^) induces matrix degradation, inflammation, and cell death (Xu et al., 2024; Zhang M. et al., 2019), whereas low fluid shear stress (2–10 dyne/cm^2^) may have a protective effect on cartilage (Chang et al., 2019; Zhao and Xia, 2024). These studies suggest that shear forces of different intensities may have different potentials to regulate cartilage metabolic homeostasis. A deeper understanding of the shear behavior of the TMJ may help to better understand why the TMJ adapts to different shear force changes. In the study on the dynamic shear properties of the TMJ condylar cartilage, it was found that the frequency and amplitude of the shear strain affected the shear behavior of the condylar cartilage (Tanaka et al., 2008a). Due to the anisotropy of collagen cross-linking, the shear resistance of the condylar cartilage is greater in the anterior-posterior direction than in the left-right direction (Tanaka et al., 2008b). This shear property suggests that the mandibular condylar cartilage can adapt to shear forces within the physiological frequency range and remodel normally and may be more susceptible to cartilage degradation under the action of abnormal medial and lateral shear stress. These findings may provide new insights for further studying the role of abnormal shear force stimulation in the pathogenesis of TMJD.

### 2.4 Enhanced adaptive remodeling capacity of young animals compared with older animals

Condylar cartilage of growing mice shows high remodeling ability. In young mice, TMJ condylar extracellular matrix collagen fibers and proteoglycan increased following the removal of aberrant occlusal stimuli or the restoration of a normal masticatory loading (Chen et al., 2009; Zhang H‐Y. et al., 2015). Reducing dietary loading can relieve cartilage degeneration by decreasing incisor cutting (Liu et al., 2014). Metal tubes were installed on the incisor teeth of growing mice to increase the vertical height of tooth occlusion so that TMJ was in a state similar to the tension effect, which could enhance the proliferation of condylar chondrocytes and significantly thicken the cartilage (Liu et al., 2019). However, in adult rats, TMJ condylar cartilage was insensitive to changes in mechanical loading (De Carli et al., 2023). With the increase in age, the remodeling ability of the TMJ seems to gradually weaken.

## 3 Pathological bone remodeling in subchondral bone

Trabecular bone is dynamically balanced between osteoblast-mediated bone production and osteoclast-mediated bone resorption when functional biomechanical stimulation is applied (Liu et al., 2021). The resorption and production of subchondral trabecular bone are out of balance under abnormal mechanical stress, primarily resulting in bone loss.

### 3.1 Asymmetric mechanical stress distribution induces unilateral bone lesion

Functional mandibular deviation can change the mechanoreceptor response characteristics of TMJ, increase the expression of mechanical response factors PRG4 and Ihh, and may also cause a change in TMJ functional loading through the overexpression of VEGF protein and oxidative stress/nitric oxide imbalance (Kokai et al., 2007; Stojić et al., 2020; Yang W. et al., 2020; Zou et al., 2022). Zhang et al. conducted an animal model of mandibular skew caused by the asymmetric traction force of a spring installed between the zygomatic arch and mandibular Angle during surgery. The results showed that the TMJ subchondral bone of the mandible on the surgical side showed obvious pathological changes, such as irregular trabecular shape, fractures, and cysts (Zhang C. et al., 2015). Similarly, some scholars observed decreased bone density and increased osteoclast activity in the subchondral bone on the side where the splint was placed (Liu et al., 2022). When the bilateral TMJ is in an asymmetric mechanical environment, pathological bone remodeling of the subchondral bone of the condyle often seems to occur on the deviated side with greater mechanical stress. Some researchers have proposed that high-amplitude compressive stress applied to the joint surface causes joint injury to start with the occurrence and propagation of micro-damage and that micro-damage to the subchondral bone can reduce bone strength and stiffness (Malekipour et al., 2020; Malekipour et al., 2022). The specific pathway from abnormal mechanical stimulation to TMJ subchondral bone injury is still unclear, and whether micro-damage is involved in subchondral bone injury may become a new research direction.

### 3.2 Decreased mechanical loading induces bone transient loss

Appropriate mechanical force stimulation is necessary for subchondral bone remodeling, and the strength of applied mechanical force affects bone microstructure. Lack of appropriate mechanical loads can lead to transient bone loss in the subchondral bone. Young mice with lower chewing functional loads showed notable reductions in subchondral bone trabecular thickness and bone volume fraction (Chen et al., 2009). In addition to supporting and absorbing strain in healthy joints, subchondral bone trabeculae are also crucial for the movement and metabolism of cartilage nutrients (Castañeda et al., 2012). Subchondral bone loss may increase the stress transmitted to the overlying cartilage, leading to secondary cartilage damage. When the functional loading was continuously reduced, linked indicators gradually restored to normal values rather than experiencing further declines. Chen hypothesized that the nuclear factor-KB(NF-KB)/RANK ligand (RANKL)/osteoprotegerin (OPG) signaling pathway might be crucial in this shift, which could be linked to the decline in osteoclast numbers. According to the experimental data, the higher OPG/RANKL ratio is thought to protect subchondral bone (Chen et al., 2009).

## 4 Metabolic disorders in TMJ disc

The disc is the main stress distribution tissue in the TMJ, providing lubrication and cushioning and reducing the load-bearing force between the condyle and the fossa during movement (Tanaka et al., 2008c). The disc can alter its shape to accommodate the dynamic close contact relationship between the condyle and the fossa during mandibular movement, as well as alter its volume by absorbing or releasing synovial fluid. The thickness of the anterior, middle, and posterior bands can vary depending on the intensity of mechanical stress stimulation (Yasuno et al., 2024; Sun et al., 2009). The biomechanical properties of the TMJ disc are associated with the composition of ECM, in which collagen fibers provide tensile stiffness for the tissue, and proteoglycans provide compressive stiffness (Kiani et al., 2002; Willard et al., 2012; Zhang H. et al., 2019). Proteoglycans are responsible for the remodeling and pathological processes of the TMJ disc in addition to preserving the tissue’s integrity.

### 4.1 Proteoglycan reduction under low mechanical loading

Kozue et al. found that the mRNA expression of glycosaminoglycan (GAG) and multiple proteoglycans decreased after rats were fed a soft diet for 4 weeks, and the disc of the soft diet group became thinner (Yasuno et al., 2024). The reduction of TMJ load caused by a soft diet seems to lead to the loss of ECM of the disc, and the thinning of the disc may be related to the loss of proteoglycan and GAG. Due to the single detection method in this experiment, more experiments such as immunohistochemical staining and Western blotting are needed in the future to provide more evidence to detect regional differences in proteoglycan expression. Nakao et al. used incisal bite to increase joint load and induce thickening of the posterior zone of the TMJ disc of rats, as well as increased expression of GAG and proteoglycans (Nakao et al., 2015). The expression of proteoglycan in TMJ discs is affected by different mechanical loading intensities. Proteoglycans may be of great significance in the adaptation of the TMJ articular disc to a complex biomechanical environment. Metal splints were placed on the maxillary and mandibular molars unilaterally for 6 weeks, and then the TMJ disc samples were compressed from 10% to 30% strain in compression tests. The results showed that the TMJ disc’s anti-compression performance, collagen, and glycosaminoglycan content remained unchanged (Henderson et al., 2015). Mechanical analysis of rodent models showed that the force on the condyle during tangential movement was greater than the force under chewing movement (Weijs and Dantuma, 1975). Incisal bite increases the frequency and duration of tangential movement, and the joint disc is subjected to greater loads than when metal splints are placed on the molars, which may be the reason for the differences in the performance and composition of the disc between the two models.

### 4.2 Abnormalities in the disc’s nutritional supply

Studies have shown that nutrients (glucose, lactate, and oxygen) provided by marginal blood vessels and synovial fluid play an essential role in intervertebral disc homeostasis by affecting cell matrix metabolism and inflammatory response (Bibby and Urban, 2004). In fibrocartilage tissue, increased compressive mechanical stress can hinder the diffusion of glucose and ions, and it may further regulate the nutritional metabolism of the TMJ disc (Wright et al., 2013; Wu et al., 2016). Glucose is required for energy metabolism and matrix production in articular disc cells, and reduced glucose supply may be linked to disc ageing and degeneration (Dong et al., 2023; Cisewski et al., 2015). Wu et al. used experiments to measure the metabolic rate of nutrients, solute diffusion rate, TMJ anatomical structure, and load-bearing area of pig TMJ discs and then developed a finite element (FE) model of human subjects based on computers. The results suggest that pathological continuous mechanical loads (such as bruxism) may hinder nutrient supply by affecting TMJ disc homeostasis (Wu et al., 2019). This provides a new method for further exploring the homeostasis and degeneration mechanism of TMJ discs related to abnormal mechanical loading. Although current animal experimental results show that an abnormal TMJ biomechanical environment can affect disc metabolism at the molecular level, pathological changes in the disc are rarely observed intuitively from tissue morphology. Structural damage to the disc tissue, such as disc perforation, leads to clinical symptoms including joint pain, intra-articular crepitus, and abnormal mandibular movements, which can severely impact both the physical and mental health of patients (Gao et al., 2023). Based on the complexity of the TMJ biomechanical environment, it is necessary to study the stress distribution of the disc during different types of mandibular movements. Clarifying the stress distribution in different areas of the disc may help provide a theoretical basis for developing new animal models of disc stress changes.

### 4.3 “Mechanical fatigue” phenomenon in the disc

A mechanical phenomenon known as “mechanical fatigue” occurs when materials or pieces gradually deteriorate and distort as a result of constant stress and strain during mechanical movement. Its influencing variables include the frequency and magnitude of mechanical loading. The process known as fatigue failure occurs when materials are harmed by alternating stress at levels well below their yield or strength limits. These two concepts are related to the degeneration and damage of synovial joints (Petersen et al., 2023; Sise et al., 2025). To measure the magnitude and frequency of mechanical load on the mandible, Nickel employed TMJ Energy Densities (ED) and jaw muscle duty factors (DF), combining the two to calculate the mechanical behaviour score (MBS). According to the findings, those with TMJ disc displacement (DD) had considerably higher ED, masseter and temporalis DF, and MBS than healthy individuals without DD (Nickel et al., 2024; Iwasaki et al., 2017). The disc tissue of TMJ DD seems to be more susceptible to mechanical fatigue. According to Schiffman’s analysis of 789 TMJ magnetic resonance images and 794 TMJ CBCT data, the majority of participants had stable and reversible TMJ illness diagnoses, with rates of soft and hard tissue progression to degenerative joint disease being 14% and 15%, respectively (Schiffman et al., 2017). Since it is currently hard to anticipate whether a TMJD diagnosis will advance or reverse over time, it is worthwhile to investigate whether a higher MBS is linked to TMJ degenerative illness.

## 5 The relationship between abnormal mechanical force-induced TMJ tissue changes and the occurrence of pain

As summarized in previous sections, abnormal mechanical stimulation can lead to TMJ cartilage degeneration and subchondral bone pathological remodeling, indicative of degenerative joint disease. TMJ structural dysfunction is often accompanied by pain in the craniofacial and orofacial regions, consequently, pain is frequently the primary reason patients seek treatment, making TMJD one of the leading causes of non-dental orofacial pain (Okeson and de Leeuw, 2011; Greene, 2010). Due to the complex, multifactorial pathogenesis of TMJD, the relationship between TMJ degenerative changes and pain remains unclear. Multiple animal models have been developed to investigate the causal relationship between TMJ structural alterations and pain onset, with mechanical loading models playing a pivotal role. Kartha et al. developed a non-invasive, adjustable mechanical loading model (load: 2N and 3.5 N) in rats to simulate acute or chronic pain in the TMJ region (Kartha et al., 2016). Compared to invasive models involving open trauma or intra-articular injection of chemicals to induce structural damage, this model generates persistent pain by modulating mandibular opening forces, better mimicking the natural pathological progression of pain development in TMJD patients and enhancing clinical relevance (Xiang et al., 2021). While both load intensities (2N and 3.5 N) induced TMJ cartilage degeneration, only the 3.5 N load group exhibited persistent pain and significant upregulation of inflammatory markers HIF-1α and TNF-α. This suggests that pain and TMJ cartilage pathology may involve distinct mechanistic drivers. Wang et al. utilized a sustained mouth-opening model in mice, which led to persistent orofacial mechanical hyperalgesia, TMJ cartilage degeneration, and inflammation, alongside activation of macrophages and microglia in the trigeminal nervous system (Wang et al., 2018). Numerous studies have demonstrated that pro-inflammatory cytokines produced by macrophages or other immune cells contribute to tissue degradation and pain (Kellesarian et al., 2016; Cairns, 2010). Further experiments by Wang et al. revealed that inhibiting macrophage and microglia activation effectively prevented orofacial hyperalgesia but did not ameliorate TMJ structural damage. These findings highlight the critical role of macrophages and microglia in TMD pain progression, suggesting their potential as therapeutic targets for TMJ-related pain. The persistence of TMJ degeneration despite pain alleviation underscores the complexity of molecular mechanisms governing TMJ degeneration and pain. Given the importance of the pericellular matrix in maintaining chondrocyte mechanical properties (Khoshgoftar et al., 2018), Franklin et al. employed adjustable mechanical loading models to investigate the relationship between varying pain levels and pericellular matrix microstructural dysregulation, and the results showed that increased expression of aggrecan degradation products in both pain-resolved and pain-persistent groups (Franklin et al., 2022), indicating that pain may not directly regulate pericellular matrix remodeling. Studies using mechanical loading models to explore TMJ pain and tissue degeneration suggest that these processes can occur in partially overlapping temporal phases. Certain molecules, such as pro-inflammatory cytokines, may simultaneously induce pain and joint degeneration. However, whether TMJ structural changes invariably lead to pain, or whether pain predicts TMJ degeneration, remains unresolved. Future studies should extend experimental timelines, incorporate graded loading intensities, and investigate long-term dynamic relationships between pain thresholds and pathological progression to clarify these interactions.

## 6 Conclusion

**FIGURE 1:**
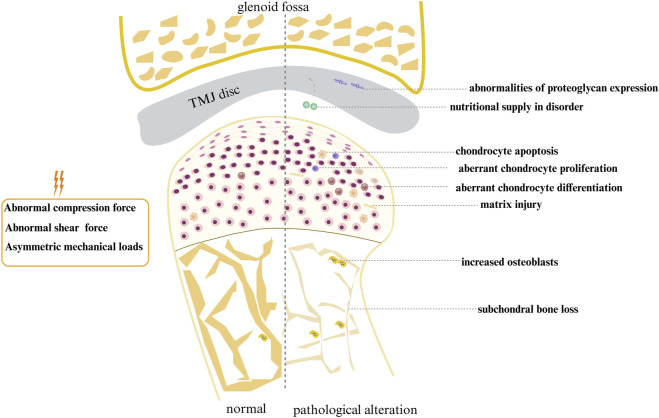
Schematic diagram of pathological changes in TMJ due to abnormal mechanical stimuli.

Abnormal mechanical stress induces degeneration of the condylar cartilage, characterized by dysregulated chondrocyte proliferation and differentiation, elevated cell apoptosis, and ECM injury. The ability of TMJ condylar cartilage to adapt to changes in the mechanical load environment for remodeling is influenced by age, type, intensity, and duration of the applied mechanical load. When aberrant mechanical force stimuli are delivered to the subchondral bone, bone loss is the predominant outcome. Variations in mechanical loads have an impact on metabolic activity and the expression of disc matrix components, as shown in Figure 1.

The intricate mechanical stress environment in the TMJ cannot be accurately simulated by current mechanical loading models. In the review, we summarized that the majority of experimental models investigating altered mechanical stress distribution in the TMJ were currently based on rodent models. However, it is imperative to acknowledge a critical limitation arising from the morphological divergence between rodent and human TMJ structures. Notably, rodents exhibit a shallower and flatter glenoid fossa with the absence of an articular eminence compared to the human TMJ architecture (Suzuki and Iwata, 2016). Such anatomical disparities fundamentally constrain the capacity of rodent models to precisely replicate human TMJ kinematic patterns and long-term mechanical loading profiles.

Nevertheless, rodent models retain indispensable value in elucidating the pathogenic mechanisms of TMJD and exploring therapeutic strategies, owing to their experimental tractability, mechanical stress controllability, and cost-effectiveness, and provide an irreplaceable platform for exploring the relationship between mechanical stress and structural changes in the TMJ. For instance, the forced mouth-opening model, which applies sustained compressive forces via a spring device, enables precise quantification of the dose-dependent relationship between mechanical load intensity and cartilage degeneration. Similarly, the asymmetric mechanical loading model allows for the differentiation of specific responses in bilateral TMJ under differential stress conditions.

When mechanical loading conditions differ from normal, a wider range of test parameters (e.g., load levels, strain rates, frequencies, etc.), together with adequate characterization of the compressive, tensile, and shear properties of TMJ tissues, may be required to identify patterns of change in future models capable of simulating realistic loading environments. A thorough understanding of the mechanical characterization of the TMJ may contribute to a better understanding of the aetiology of TMJ degeneration.
